# Fishing for cures: The alLURE of using zebrafish to develop precision oncology therapies

**DOI:** 10.1038/s41698-017-0043-9

**Published:** 2017-11-27

**Authors:** Matteo Astone, Erin N. Dankert, Sk. Kayum Alam, Luke H. Hoeppner

**Affiliations:** The Hormel Institute, University of Minnesota, Austin, MN 55912 USA

## Abstract

Zebrafish have proven to be a valuable model to study human cancer biology with the ultimate aim of developing new therapies. *Danio rerio* are amenable to in vivo imaging, high-throughput drug screening, mutagenesis, and transgenesis, and they share histological and genetic similarities with *Homo sapiens*. The significance of zebrafish in the field of precision oncology is rapidly emerging. Indeed, modeling cancer in zebrafish has already been used to identify tumor biomarkers, define therapeutic targets and provide an in vivo platform for drug discovery. New zebrafish studies are starting to pave the way to direct individualized clinical applications. Patient-derived cancer cell xenograft models have demonstrated the feasibility of using zebrafish as a real-time avatar of prognosis and drug response to identify the most ideal therapy for an individual patient. Genetic cancer modeling in zebrafish, now facilitated by rapidly evolving genome editing techniques, represents another innovative approach to recapitulate human oncogenesis and develop individualized treatments. Utilizing zebrafish to design customizable precision therapies will improve the clinical outcome of patients afflicted with cancer.

## Introduction

Precision medicine in oncology arises from recognition that patient-specific clinical, genetic, and molecular features dictate effectiveness of a given treatment. Therefore, precision oncology seeks to identify the most effective therapy for an individual patient, based on characterization of their cancer. The development of genomic technologies and molecular diagnostics enables detection of cancer biomarkers. These relevant abnormalities associated with specific cancers lead to the identification of actionable targets. Diagnostic (associated with the presence of a specific pathophysiological state), prognostic (associated with disease outcome), and predictive (associated with drug response) cancer biomarkers guide clinical treatment decisions and direct the use of drugs that modulate the activity of the specific actionable target.^1,2^

Zebrafish (*Danio rerio*) have rapidly emerged as a promising animal model of human cancer. Histological, molecular, and genetic similarities to *Homo sapiens* facilitate zebrafish studies of human malignancies. Zebrafish are amenable to in vivo fluorescent imaging, chemical and genetic screens, transgenesis, and high-throughput mutagenesis assays, which have brought zebrafish to the center stage of future advances in the field of precision oncology.^3^ A variety of attributes have contributed to the emergence of zebrafish as an attractive vertebrate model organism. Zebrafish are easy and inexpensive to maintain and breed with high fecundity, which facilitates large studies and high-throughput in vivo assays. Another advantage of working with zebrafish embryos is their conserved vertebrate features develop rapidly and genetic studies restricted to embryos can be completed in days to weeks rather than weeks to months as is often the case with mammalian models.^3,4^ However, it is important to note that zebrafish sexual maturation takes about three months, so generation studies (i.e., transgenics, knockouts, etc.) require a similar time frame as murine models. The small size, external development, and transparency of zebrafish embryos make them amenable to fluorescent live imaging to monitor physiological processes (e.g., development, morphogenesis, angiogenesis, etc.) and pathological phenomena (e.g., cancer initiation, tumorigenesis, metastasis, etc.). Taken together, the attractive features of the zebrafish model system underscore the reasons it has gained prominence in the study of cancer and serves as an excellent addition to other common oncology models and platforms.

This review aims to provide an overview of how current zebrafish cancer studies lay the groundwork for utilization of this model organism in precision oncology, highlighting specific studies oriented to the development of zebrafish-based patient-specific approaches for cancer treatment. The challenges and shortcomings of zebrafish cancer studies are presented as areas of the field requiring advancements and growth.

## Zebrafish: From modeling classic cancer research to precision oncology

The application of the zebrafish model to precision oncology remains in its infancy, and there are not yet examples of direct use of zebrafish to guide patient-specific cancer treatments in the clinic. However, the field has matured enough to move toward this aim in the near future. Modeling cancer in zebrafish has provided important insights that contribute to the development of precision oncology as well as straightforward examples of advantages and feasibility of direct clinical utilization (Table 1).

**Table 1:** The contribution of zebrafish cancer models to precision oncology

Cancer type	Model	Transgene/injected cells	Approach	Results	Specific contribution to precision oncology	Ref.
Melanoma	Transgenic line	*mitfa:HRAS* ^*G12V*^	Pharmacological test in vivo	Small molecule inhibitors of MEK and PI3K/mTOR suppress the melanocyte hyperplasia phenotype.	In vivo validation of targeted drugs for the treatment of melanoma.	25
	Transgenic line	*mitfa:HRAS* ^*G12V*^	In vivo drug screening	Two FDA-approved compounds cooperate with MEK inhibitors to suppress the growth of transformed melanocytes.	Discovery of two new potential drugs for the treatment of melanoma.	25
	XT	Human uveal melanoma cells generated from primary tumors and metastasis	Pharmacological test in vivo	Targeted inhibition of known pathways by specific drugs is effective in counteracting cancer cells migration and proliferation.	Validation of a zebrafish xenograft model as a drug screening platform for the treatment of melanoma.	66
Glioma	Transgenic line	*ptf1a:Gal4;UAS:GFP-UAS:DAAkt1*	Pharmacological test in vivo	AKT1/2 inhibitor suppresses gliomagenesis, inhibits cellular proliferation, and induces apoptosis in established gliomas.	In vivo identification of a targeted drug for the treatment of glioma.	28
	XT	Human glioblastoma cells	Pharmacological test in vivo	JNK, ERK, and PI3K inhibitors suppress angiogenesis induced by glioblastoma cells.	In vivo validation of targeted drugs for the treatment of glioblastoma via angiogenesis inhibition.	73
	XT	Human glioblastoma cells	In vitro drug screening in vivo pharmacological test	A novel small molecule radiation sensitizer enhances the tumor growth-inhibitory effects of ionizing radiation.	Discovery of a new small molecule radiation sensitizer for the treatment of glioblastoma.	74
	XT	Glioma stem cells (GSCs) isolated from a human glioblastoma cell line	Pharmacological test in vivo	A synthetic compound, Nordy, suppresses angiogenesis, tumor invasion, and proliferation of the zebrafish GSC xenograft.	In vivo validation of a drug targeting GSCs for the treatment of glioblastoma.	75,76
	XT	Human glioblastoma cells	Pharmacological test in vivo	A drug with a known anti-cancer effect in cell culture inhibits proliferation and invasion in the xenograft model.	Proof of principle for the use of a zebrafish orthotopic xenograft model as a drug screening platform for the treatment of glioblastoma.	60
	XT	Patient-derived glioma cells	Pharmacological test in vivo	Currently used glioblastoma therapeutics decrease xenotransplant tumor burden and significantly rescue survival.	Validation of a zebrafish orthotopic xenograft model as a drug screening platform for the treatment of glioblastoma.	9
Brain pediatric tumors	XT	Mouse ependymoma, glioma, and choroid plexus carcinoma cells	Pharmacological test in vivo	A cytotoxic chemotherapeutic agent (5-fluorouracil) and a tyrosine kinase inhibitor suppress ERBB2-driven gliomas.	Proof of principle for the use of a zebrafish orthotopic xenograft model as a drug screening platform for the treatment of pediatric brain tumors.	71
Pancreatic cancer	XT	Human pancreatic adenocarcinoma cells	Pharmacological test in vivo	A known small molecule inhibitor, U0126, targeting the KRAS signaling pathway, represses proliferation and migration of cancer cells.	In vivo validation of a targeted drug for the treatment of pancreatic cancer.	77
Leukemia and lymphoma	WT embryos	—	In vivo drug screening	Chemicals that enhance prostaglandin (PG) E2 synthesis increase HSC numbers.	Development of Prohema, currently in Phase II clinical trials for use in leukemia and lymphoma patients receiving blood transplantations.	47,48
T-ALL	XT	Patient-derived T-ALL cells	Pharmacological test in vivo	A bone marrow sample derived from a T-ALL patient harboring a *NOTCH1* mutation responds to NOTCH1 inhibitor in the zebrafish xenograft model.	Proof of principle for the use of a zebrafish xenotransplantation model as a preclinical platform for a personalized therapy.	5
Thyroid cancer	Transgenic line	*tg:BRAF* ^*V600E*^	Pharmacological test in vivo	Combinatorial treatment with BRAF and MEK inhibitors rescue normal follicular architecture, restore thyroid hormone production, and reduce epithelial mesenchymal transition.	In vivo validation of targeted drugs for the treatment of thyroid cancer.	49
Hepatocellular carcinoma	Transgenic line	*fabp10a:pt-β-catenin*	In vivo drug screening	Two c-Jun N-terminal kinase (JNK) inhibitors and two anti-depressants suppress β-catenin-induced liver growth.	Discovery of two classes of potential targeted drugs for the treatment of hepatocellular carcinoma.	56
Retinoblastoma	XT	Human retinoblastoma cells	Pharmacological test in vivo	Orthotopic xenograft of retinoblastoma cells permits quantitative analysis of cancer cells proliferation and the anti-cancer effect of drugs systemically administered.	Validation of a zebrafish orthotopic xenograft model as a drug screening platform for the treatment of retinoblastoma.	62
Pancreatic ductal adenocarcinoma (metastasis)	XT	Pancreatic carcinoma cells and fragments of resected tumor tissue	Pharmacological test in vivo	miR-10A suppression by knockdown or retinoid acid receptor antagonists blocks metastasis.	In vivo validation of a new molecular target and anti-metastatic targeted drugs for the treatment of pancreatic cancer.	8
Prostate cancer (metastasis)	XT	Human prostate cancer cells	Pharmacological test in vivo	Pharmacologic inhibitors of SYK kinase, currently in phase I–II trials for other indications, prevent metastatic dissemination.	In vivo validation of anti-metastatic targeted drugs for the treatment of prostate cancer.	88
	XT	Human prostate cancer cells	Pharmacological test in vivo	The small molecule VPC-18005, targeting ERG, exhibits anti-metastatic activity against prostate cancer cells aberrantly expressing ERG.	In vivo validation of an anti-metastatic targeted drug for the treatment of prostate cancer.	89
Melanoma (metastasis)	XT	Mouse melanoma cells	Pharmacological test in vivo	The FDA-approved anti-DNA virus agent cidofovir inhibits metastasis of FGF2-driven tumor cells.	In vivo validation of an anti-metastatic targeted drug for the treatment of melanoma.	90
Breast cancer (metastasis)	XT	Triple-negative breast cancer cells	Pharmacological test in vivo	Specific inhibition of Arf1 by small molecule LM11 impairs metastatic capability of breast cancer cells.	In vivo validation of a potential anti-metastatic precision oncology treatment for breast cancer patients with ARF1 amplification.	91
	XT	Triple-negative breast cancer cells	Pharmacological test in vivo	Novel compounds designed to antagonize P2 × 7 receptor inhibit invasion of breast cancer cells.	In vivo validation of anti-metastatic targeted drugs for the treatment of breast cancer.	92
	XT	Triple-negative breast cancer cells	Pharmacological test in vivo	Inhibition of signaling between human CXCR4 and zebrafish ligands by the small molecule IT1t impairs breast cancer early metastases.	In vivo validation of an anti-metastatic targeted drug for the treatment of breast cancer.	82
	XT	Primary culture of breast cancer bone metastasis	Xenograft and imaging	Transplanted primary cell behavior reflects the clinical course of the patient’s medical history.	Proof of principle for the use of zebrafish xenograft for the evaluation of cancer patient prognosis.	7
Ewing sarcoma (metastasis)	XT	Human Ewing sarcoma cells	Pharmacological test in vivo	The SIRT1/2 inhibitor Tenovin-6 prohibits tumor growth and spread of cancer cells.	In vivo validation of a new molecular target and an anti-metastatic targeted drug for the treatment of Ewing sarcoma.	93
Gastrointestinal tumors (metastasis)	XT	Tumor explants from pancreas, colon, and stomach carcinoma	Xenograft and imaging	Xenografts of primary human tumors show rapid invasiveness and micrometastasis formation after transplantation in the yolk or organotopically in the liver.	Validation of a zebrafish xenotransplantation model as a platform for the analysis of metastatic behavior of primary human tumor specimen.	6

A summary of studies described in this review that exemplify the utility of zebrafish cancer models in precision oncology research. Contributions of each model to the precision oncology field have been highlighted

*XT* xenotransplantation, *T-ALL* T-cell acute lymphoblastic leukemia

**Fig. 1:**
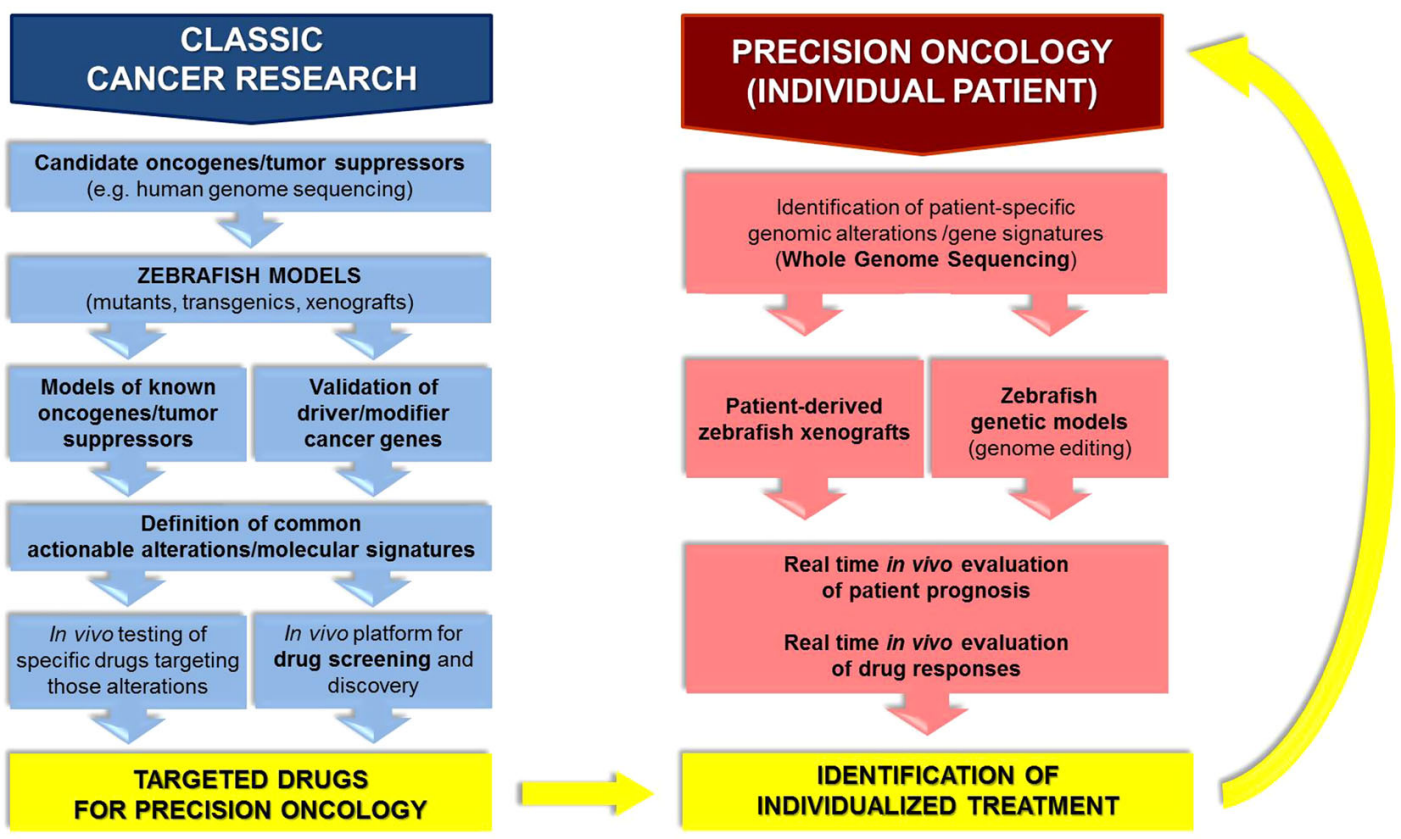
Applications of the zebrafish model in precision oncology. Classic cancer research using zebrafish has contributed to precision oncology through the establishment of numerous cancer models, leading not only to significant advancements in cancer biology, but also to the definition of targeted drugs suitable for personalized cancer treatments (blue, left). Possible applications of zebrafish in the clinic to drive personalized therapies for specific patients have also been shown. The feasibility of this approach has been demonstrated through the use of patient-derived zebrafish xenografts and generation of transgenic zebrafish modeling mutations or translocations defining a specific patient’s tumor (red, right)

Classic cancer modeling via mutagenesis, transgenesis, and xenotransplantation has contributed in numerous ways to precision oncology (Fig. 1, left). Zebrafish cancer models have facilitated (i) the identification and in vivo validation of molecular players in tumorigenesis and metastasis, (ii) the definition of actionable alterations and therapeutic targets, and (iii) the discovery of tumor biomarkers and genetic signatures as potential diagnostic and prognostic indicators. Moreover, several studies have exemplified the potential of zebrafish models to contribute more significantly and directly to precision oncology through (iv) identifying and testing drugs for targeted inhibition of specific pathways/alterations by utilizing zebrafish as an in vivo drug screening platform. A number of small molecules that might represent new targeted drugs for individualized medicine have been identified through this approach. Notably, the rapidly increasing number of patient-derived cancer cell xenografts^5–10^ places zebrafish on the road toward its clinical application for the treatment of individual cancer patients. Various studies have demonstrated the applicability of these models in (v) evaluating patient prognosis in vivo and (vi) directing individualized treatments in real-time based on responses to drugs of patient cancer cell xenografts (Fig. 1, right). Taken together, modeling cancer in zebrafish has evolved to the extent that precision oncology applications are emerging.

## Genetic models of cancer

To date, innumerable zebrafish genetic models of cancer have been generated, and the number continues to rapidly increase. Genetic cancer models have been developed using various strategies, including transient, stable, and double transgenesis and various inducers of mutagenesis (Table 2). Their use in precision oncology is gaining momentum. Here, we will discuss the most significant reports exemplifying this evolution.

**Table 2:** Genetic models of cancer

Cancer type	Genetic system	Transgenes/mutated genes	Ref.
Melanoma	Transgenic line	*mitfa:BRAF* ^*V600E*^; p53 mutant background	14
Melanoma	Transgenic line	*mitfa:NRAS* ^*Q61K*^; p53 mutant background	16
Melanoma	Transgenic line	*mitfa:HRAS* ^*G12V*^	17
Melanoma	Transgenic line	*kita:HRAS* ^*G12V*^	18
Melanoma	Transgenic line	*mitfa:GNAQ* ^*Q209P*^; p53 mutant background	19
Brain tumors	Transgenic line	*krt5:Gal4VP16; UAS:mCherry-KRAS* ^*G12V*^	27
Brain tumors	Transgenic line	*gfap:Gal4VP16; UAS:mCherry-KRAS* ^*G12V*^	27
Glioma	Transgenic line	*ptf1a:Gal4;UAS:GFP-UAS:DAAkt1*	28
MPNST	Mutant lines	Heterozygous mutations in 11 ribosomal protein genes	31
MPNST	Deletion	15.2 Mb deletion in chromosome 1	32
MPNST	Mutant line	*tp53* ^M214K^	33
Neurofibromas/MPNST	Mutant lines	*mlh1* ^*−/−*^, *msh6* ^*−/−*^, *msh2* ^*−/−*^	34
MPNST	Mutant lines	Heterozygous mutations in 17 ribosomal protein genes	35
Pancreatic cancer	Transgenic line	*ptf1a:Gal4-VP16; UAS:mutated KRAS*	36,38–40
T-ALL	Transgenic line	*rag2:mMyc*	44
T-ALL	Transgenic line	*hsp70:Cre; rag2:lox-dsRED2-lox-EGFP-mMyc*	45
T-ALL	Transgenic line	*rag2:ICN1-EGFP*	46
Thyroid cancer	Transgenic line	*tg:BRAF* ^*V600E*^	49
Hepatocellular carcinoma	Transgenic line	Mifepristone-induced Cre-mediated recombination: *fabp10:loxP-mCherry-loxP-EGFP- kras* ^*V12*^	55
Hepatocellular carcinoma	Transgenic line	*fabp10a:pt-β-catenin*	56
Colon adenoma	Mutant line	*apc* ^*mcr*^ mutant injected with mRNA encoding oncogenic *V5-KRAS* ^*G12D*^	98

Significant zebrafish genetic cancer models, including all those discussed in the review, have been summarized

*MPNST* malignant peripheral nerve sheath tumors, *T-ALL* T-cell acute lymphoblastic leukemia

### Melanoma

Melanoma research offers many concrete examples of genetic zebrafish models used for the definition of new therapeutic targets and as an in vivo platform for drug screening. Melanoma accounts for the death of over 70% of skin cancer patients and only 14% of patients with metastatic disease survive for five years. Unlike many other tumor types, new cases and mortality of melanoma are still rising.^11,12^ While some oncogenic driver mutations, such as *BRAF* and *NRAS*, have been identified in melanoma, the efficacy of therapies is limited and the prognosis of metastatic melanoma patients remains poor.^13^ Many spontaneous, oncogene-driven zebrafish models of melanoma exist. In 2005, Patton et al.^14^ expressed BRAF^V600E^ in melanocytes using the microphtalmia-associated transcription factor a (*mitfa*) promoter. These fish developed nevi, but required a p53^M214K^ mutant zebrafish background for melanoma development in ~5% of zebrafish by four months.^14^ A *crestin*:EGFP reporter, recapitulating the embryonic neural crest expression patter of *crestin*, showed that a fate change occurs at melanoma initiation in this model, as a single melanocyte reactivates the neural crest progenitor state.^15^ Similarly, human oncogenic NRAS^Q61K^ expression under the control of *mitfa* promoter resulted in a transgenic fish that required p53 loss of function for the genesis of melanoma.^16^ The first p53 mutation-independent model was developed through expression of human oncogenic HRAS^G12V^ driven by the same *mitfa* promoter fragment. In this model, however, melanoma does not arise at a high frequency and takes several months to develop.^17^Instead, when HRAS^G12V^ expression is driven in melanocyte progenitor cells by the *kita* (*c-kit* in humans) promoter, melanoma occurs spontaneously by 1–3 months in ~20% of fish.^18^ Recently, a novel zebrafish transgenic model of uveal melanoma was created by expressing oncogenic GNAQ^Q209P^ in the melanocyte lineage using again the *mitfa* promoter. The corresponding p53 inactivation was also required for the malignant progression in this system.^19^ Importantly, zebrafish models of melanoma closely resemble human cancer, both in terms of histopathological features and molecular signatures.^20^

These models have confirmed the role of relevant oncogenes in melanoma genesis and progression. Moreover, they have proven to be outstanding tools to test and screen for other genes that promote melanoma onset and might represent new therapeutic targets, and even, in the near future, tumor biomarkers for personalized cancer therapy. An excellent example has been reported by Ceol and colleagues. They have used transgenic zebrafish overexpressing BRAF^V600E^ on a p53 mutant background to test genes in a recurrently amplified region on chromosome 1. The histone methyltransferase SETDB1 has been found to cooperate with BRAF^V600E^ and accelerate melanoma. Its relevance in human malignant melanoma has also been demonstrated, and therefore, SETDB1 has been revealed as a novel oncogene in melanoma.^21^ RAC and RSK1, whose hyperactivation has been detected in human melanoma, have been shown in distinct studies to contribute to melanoma progression when constitutively activated in *mitfa*:HRAS^G12V^ and *mitfa*:BRAF^V600E^;p53^−/−^ transgenic backgrounds, respectively.^22,23^ Lister et al.^24^ have used a temperature-sensitive *mitfa* mutant to show the oncogenic activity of Mitfa transcription factor in BRAF^V600E^ transgenic zebrafish and the regression of BRAF^V600E^ *mitfa* melanoma after Mitfa activity abrogation, thus presenting Mitfa as a promising therapeutic target. The use of zebrafish to identify novel oncogenes begins to exemplify how this model organism will be utilized to overcome tumor heterogeneity through precision oncology.

The significance of zebrafish melanoma models in translational medicine and precision oncology is not limited to the discovery and characterization of potential therapeutic targets, as various studies have already shown the efficacy of zebrafish in identifying, discovering, and testing drugs for the development of new melanoma treatments. Small molecule inhibitors of MEK and PI3K/mTOR, known players in melanoma, have been validated in vivo as targeted drugs suppressing melanocyte hyperplasia phenotype in HRAS^G12V^ transgenic embryos.^25^ Moreover, a zebrafish screen of FDA-approved compounds led to the discovery of two new potential drugs cooperating with MEK inhibitors to suppress the growth of transformed melanocytes.^25^ Transgenic *mitfa*:BRAF^V600E^;p53^−/−^ zebrafish embryos demonstrate a gene signature enriched for markers of multipotent neural crest cells. A chemical genetic screen was, therefore, performed to identify small molecule suppressors of the neural crest lineage. A positive result was obtained with the inhibitors of dihydroorotate dehydrogenase, whose activity as an anti-melanoma agent was then confirmed in vitro and through mouse xenograft models.^26^ To fully realize the utility of zebrafish in precision oncology, translating these types of drug identification and validation studies to a patient sample size of one is the ultimate goal, such that treatments can be tailored to the individual patient based on zebrafish surrogates of the individual’s tumor.

### Neurological tumors

Neurological tumors have also been modeled via transgenic expression of oncogenes, demonstrating the potential to define relevant actionable alterations driving cancer progression and to successfully test specific drugs targeting those alterations.^27,28^ The focus of most zebrafish studies on brain tumors is malignant glioma, which accounts for 70% of malignant primary brain tumors, and in particular glioblastoma, the most aggressive primary brain cancer, accounting for 70% of malignant gliomas.^29,30^ Transgenic models of malignant peripheral nerve sheath tumors have also been described.^27,31–35^ Jung and colleagues established a transgenic zebrafish that overexpressed dominant active, human AKT1 at the *ptf1a* domain leading to gliomagenesis. Pharmacological tests identified AKT1/2 inhibitor as a targeted drug capable of effectively suppressing gliomagenesis, inhibiting cellular proliferation, and inducing apoptosis in established gliomas.^28^ The scope of available brain tumor models offers promise for using zebrafish to tailor specific treatment approaches to individual neurological cancer patients.

### Pancreatic cancer

The Gal4/UAS transgenic system, based on the ability of the Gal4 transcriptional activator to drive the expression of multiple transgenes under the regulation of UAS (upstream activator sequence) regulatory elements, is widely used to model KRAS-initiated pancreatic cancer in zebrafish.^36^ Pancreatic cancer is a deadly genetic disease, with a dismal ~9% five year survival rate.^37^ The majority of pancreatic cancers are pancreatic ductal adenocarcinomas (PDACs) and over 90% of them carry an activating point mutation in the *KRAS* gene.^36^ Genetic models based on the Gal4/UAS system enable assessment of the effects of different *KRAS* mutations and the ability of other proteins to alter the response to oncogenic KRAS, potentially leading to the identification of new targets for precision oncology therapeutic strategies.^38,39^ In this regard, the involvement of a variety of core signaling pathways, including TGFβ, Wnt, Notch, and Hedgehog, in pancreatic cancer development has also been investigated using Gal4/UAS system.^36,38,40^

### Leukemia

Leukemia, the ninth most common cancer type is a cancer of blood-forming tissues usually involving dysfunction of white blood cells.^41^ Leukemia has been modeled mainly through transgenesis. A zebrafish model of T-cell acute lymphoblastic leukemia (T-ALL), the most common type of childhood leukemia,^42,43^ was created in the early 2000s expressing a mouse *c-Myc* transgene fused to green fluorescent protein (GFP) under the control of a zebrafish *rag2* promoter.^44^ Visualization of GFP^+^ leukemic cells has demonstrated leukemia originates in the thymus, disseminates to the gill arches and surrounding retro-orbital soft tissue, and then spreads to skeletal muscle and abdominal organs.^44^ Feng and colleagues have subsequently improved this model by developing conditional, heat-inducible activation of the *c-Myc* oncogene resulting in greater penetrance of T-ALL and increased control of disease onset.^45^ Similarly, another zebrafish model of T-ALL has been created by expressing the truncated human NOTCH1 protein fused to EGFP (ICN1-EGFP) under the control of the zebrafish *rag2* promoter.^46^ While these transgenic zebrafish developed T-ALL by 5 months of age, onset of leukemia was dramatically accelerated when crossed to zebrafish overexpressing anti-apoptotic protein, Bcl2. The oncogenic synergy between NOTCH1 and Bcl2 in this model suggests genetic modifier screens may reveal other genes that interact with NOTCH1 to promote T-ALL.^46^ All of these *rag2* promoter-driven transgenic zebrafish models are amenable to drug and genetic screening to identify individualized treatment strategies for leukemia and lymphoma patients. Indeed, a zebrafish screen for therapeutics that alter the number of hematopoietic stem cells (HSCs) has led to the development of Prohema, a derivative of prostaglandin E2 (PGE2), currently in Phase II clinical trials for use in leukemia and lymphoma patients receiving blood transplantations.^47,48^

### Thyroid cancer

Stable transgenic expression of oncogenic BRAF (BRAF^V600E^) in thyroid epithelial cells has recently been shown to induce thyroid cancer in adult zebrafish. Combinatorial treatment with BRAF and MEK inhibitors rescue normal follicular architecture, restore thyroid hormone production, and reduce epithelial mesenchymal transition stimulated by BRAF^V600E^. The model has demonstrated in vivo the genetic requirement for Twist expression downstream of BRAF^V600E^, as ablation of *twist3* by CRISPR-Cas9 suppressed BRAF-mediated oncogenesis.^49^ The in vivo validation of targeted molecular therapies for the treatment of thyroid cancer demonstrates the applicability of the zebrafish system to precision oncology approaches.

### Liver cancer

Liver cancer is the second leading cause of cancer-related death. Hepatocellular carcinoma (HCC) accounts for 90–95% of liver cancer cases.^50^ Several zebrafish models of liver cancer have been developed utilizing different expression systems (reviewed well by Lu et al.^50^), including extensive contributions by Dr. Gong’s group.^51–55^ As a recent example, they developed a transgenic system for a liver-specific, mifepristone-inducible expression of oncogenic kras^V12^ via permanent genomic recombination mediated by the Cre-loxP system,^55^ which will facilitate the study of liver tumors that originate from a single cell or a small number of precursor cells through clonal expansion. In all, 20–40% of HCC are defined by an activating mutation in the gene encoding β-catenin. Evason and colleagues created a transgenic zebrafish expressing hepatocyte-specific activated β-catenin. They used the model to screen for druggable pathways that mediate β-catenin-induced liver growth and identified two c-Jun N-terminal kinase (JNK) inhibitors and two anti-depressants as potential targeted therapeutics.^56^ As is true in other tumor types discussed in this section, zebrafish have contributed to drug development to treat liver cancer.

## Transplantation cancer models

With the first experiment reported in 2005,^57^ xenotransplantation of human cells into zebrafish represent a young frontier in zebrafish cancer modeling. However, the field has evolved rapidly, and xenograft zebrafish models utilizing various injection sites, developmental stages, and transplanted specimens (i.e., human cell lines, patient-derived primary cancer cells, patient-derived tumor tissue explants) have been developed^58,59^ (Table 3). Engraftment of a diverse range of human, murine, and zebrafish tumor cells has been demonstrated. Zebrafish transplantation models offer the possibility to study many hallmarks of cancer and steps of cancer progression, such as self-renewal, tumor-induced angiogenesis, invasion and dissemination, interaction between tumor and host, and drug responses.^58,59^ Cancer specimen transplantation into embryos is certainly the most commonly used zebrafish developmental stage for undeniable advantages, including the ease of producing and injecting many embryos in a short amount of time. Furthermore, the immature state of the immune system of embryos avoids the requirement of immune suppressing agents or irradiation.^58,59^ Fluorescently labeled tumor cells have been transplanted at developmental stages varying from the blastula stage to 72 h post fertilization (hpf) in injection sites such as blastodisc, yolk sac, bloodstream, perivitelline space, and orthotopic sites, including the hindbrain ventricle and vitreous cavity.^9,58–62^ The transplanted cells can be studied for up to 21 days post fertilization (dpf), at which point the zebrafish has developed a fully functional innate and adaptive system.^59^ While embryonic xenotransplantation offers numerous advantages, a limitation is that many of the tumor types being modeled occur predominantly in adults.

**Table 3:** Transplantation cancer models

		Cancer type	Transplanted cells	Developmental stage	Injection site	Ref.
Transplantation cancer models	Cell lines	Various types	Several human cancer cells	25–35 dpf	Peritoneal cavity	63
		Uveal melanoma	Human uveal melanoma cells generated from primary tumors and metastasis	48 hpf	Yolk sac	66
		Cutaneous melanoma	Human cutaneous melanoma cells	3 hpf (blastula)	Early embryo	67
		Melanoma	Human melanoma cells derived from metastatic melanoma lesions	48 hpf	Pericardium	68
		Melanoma	Zebrafish melanoma cells	Adult	Peritoneal cavity	69
		Brain pediatric tumors	Mouse glioblastoma cells, mouse ependymoma cells, mouse choroid plexus carcinoma cells	30 dpf	Into the cerebrum via the intranasal route	71
		Vestibular schwannoma	Mouse schwannoma cells	12–48 hpf	Yolk sac	72
		Glioblastoma	Human glioblastoma cells	48 hpf	Yolk sac	73
		Glioblastoma	Human glioblastoma cells	3.5–4.5 hpf (blastula)	Blastodisc or yolk sac	74
		Glioblastoma	Glioma stem cells isolated from a human glioblastoma cell line	48 hpf	Yolk sac	75,76
		Glioblastoma	Human glioblastoma cells	48 or 72 hpf	Hindbrain ventricle	60
		Pancreatic cancer	Human pancreatic adenocarcinoma cells	48 hpf embryos and 6 mpf adults	Perivitellin cavity (embryos) and cardiac chamber (adults)	77
		Retinoblastoma	Human and mouse retinoblastoma cells	48 hpf	Vitreous cavity	61
		Retinoblastoma	Human retinoblastoma cells	48 hpf	Vitreous cavity	62
	Patient-derived cells	Glioblastoma	Patient-derived glioma cells (serum-grown adherent cells and neurospheres)	36 hpf	Midbrain-hindbrain boundary	9
		T-ALL	Leukemia cells and patient-derived leukemia cells	48 hpf	Yolk sac	5
Transplantation metastasis models	Cell lines	Various types	Human breast adenocarcinoma, fibrosarcoma and colon adenocarcinoma cells	48 hpf	Pericardium	83
		Pancreatic cancer	Human renal cell adenocarcinoma cells and human pancreatic adenocarcinoma metastasis cells	72 hpf	Pericardium	84
		Lung adenocarcinoma	Human lung adenocarcinoma cells	72 hpf	Pericardium	85
		T-ALL	Zebrafish lymphoma cells	Adult	Intraperitoneally	86
		Pancreatic ductal adenocarcinoma	Human primary pancreatic adenocarcinoma cells	48 hpf	Yolk sac	87
		Prostate cancer	Human prostate cancer cells	48 hpf	Yolk sac	88
		Prostate cancer	Human prostate cancer cells	48 hpf	Yolk sac	89
		Melanoma	Mouse melanoma cells	48 hpf	Duct of Cuvier	90
		Triple-negative breast cancer	Triple-negative breast cancer cells	48 hpf	Perivitellin cavity	91
		Triple-negative breast cancer	Triple-negative breast cancer cells	48 hpf	Yolk sac	92
		Triple-negative breast cancer	Triple-negative breast cancer cells	48 hpf	Duct of Cuvier	82
		Ewing sarcoma	Human Ewing sarcoma cells	48 hpf	Yolk sac	93
	Patient-derived cells	Breast cancer	Cultured circulating tumor cells isolated from the blood of a metastatic breast cancer patient	72 hpf	Pericardium	10
		Various types	Mouse mammary epithelial cells transformed with oncogenic Ras and tumor explants from pancreas, colon and stomach carcinoma	48 hpf	Yolk sac	6
		Pancreatic ductal adenocarcinoma	Pancreatic carcinoma cells and fragments of resected tumor tissue	48 hpf	Yolk sac	8
		Breast cancer	Breast cancer cells and primary culture of breast cancer bone metastasis	48 hpf	Duct of Cuvier	7

The zebrafish transplantation cancer and metastasis models discussed in the review have been outlined

*T-ALL* T-cell acute lymphoblastic leukemia

Xenotransplantation in juvenile and adult zebrafish seeks to overcome the limitation of translating embryonic zebrafish models to mature human cancer patients. Transplantation of human cancer cells in 30 dpf zebrafish has been established by Stoletov and colleagues in 2007 by injecting cells into the peritoneal cavity and treating the fish with dexamethasone to prevent rejection.^63^ The study of cancer cell transplantation in adult fish requires immune suppression by irradiation or dexamethasone pre-conditioning, and the use of transparent transgenic zebrafish allows the rapid identification of the transplanted cells.^58,59^ Casper fish, a cross between the nacre and roy mutant lines, lacking all types of pigments, are commonly used for this purpose.^64^ More recently, Tang et al.^65^ developed an optically clear immunocompromised transgenic mutant zebrafish line for optimized cell transplantation and direct visualization of fluorescently labeled cancer cells in the adult fish. Although juvenile and adult zebrafish transplantation models more closely match the developmental state and age of humans afflicted with cancer, the requirement for immune-deficient zebrafish represents the downside.

### Melanoma

Both embryonic and adult zebrafish have been used as transplantation models to study drug efficacy in melanoma. van der Ent and colleagues injected different human uveal melanoma cell lines generated from primary tumors and metastases into the yolk of 48 hpf zebrafish embryos. They have shown that targeted inhibition of known pathways by specific drugs proves effective in counteracting cancer cells migration and proliferation, thus demonstrating the applicability of the zebrafish xenograft model for drug screening and discovery.^66^ Other xenograft zebrafish models have been used to explore relevant pathways in melanoma, such as Nodal in cellular plasticity and tumorigenicity,^67^ and TGF-β in cellular resistance to MEK inhibitors.^68^ Recently, a drug treatment system for the long-term administration of anti-melanoma drugs in adult casper zebrafish transplanted with a zebrafish melanoma cell line has been developed. The adult environment may be relevant to test and identify new promising treatments.^69^

### Neurological tumors

A variety of zebrafish neurological tumor xenograft models have been established, primarily through xenotransplantation of different human malignant glioma cells (reviewed in Vittori et al.^70^). However, several studies have transplanted cells from mouse models of pediatric brain tumors and vestibular Schwannoma.^71,72^ A glioblastoma xenograft model has been proposed as a system for high-throughput screening of anti-angiogenic compounds. Transplanted cancer cells have been shown to be capable of inducing angiogenesis, which was enhanced by TGF-β1 and inhibited by targeted drugs, such as JNK, ERK, and PI3K inhibitors.^73^ Another zebrafish glioma xenograft model has been used to evaluate the in vivo efficacy of a novel small molecule radiation sensitizer identified through an in vitro drug screening in human glioma cells.^74^ The biological behavior of glioma cancer stem cells (GSCs) has also been explored in a zebrafish xenograft model. GSCs induced angiogenesis, which was inhibited by several anti-angiogenic agents. Moreover, the model revealed the in vivo activity of a synthetic compound, Nordy, previously found to promote GSCs differentiation in vitro. Nordy suppressed angiogenesis, tumor invasion, and proliferation of the zebrafish GSC xenograft.^75,76^ Taken together, zebrafish glioblastoma and glioma xenograft models have proven to be valuable systems for testing various molecularly targeted therapies.

Many brain tumor xenograft models have been obtained by orthotopically implanting cancer cells in embryonic or even larvae and juvenile brain.^70^ Direct transplantation into zebrafish brain facilitates cancer cell survival and proliferation, which leads to more relevant results. Glioblastoma cells have been injected in the hindbrain ventricle at 48–72 hpf to develop a xenograft assay to discover and prioritize compounds impacting glioblastoma progression. The utility of the assay was demonstrated by the ability of a drug with a known anti-cancer effect in cell culture to inhibit proliferation and invasion in the xenograft model.^60^ Another study described a platform to study the efficacy of drugs for the treatment of pediatric brain tumors. Mouse ependymoma, glioma, and choroid plexus carcinoma cells were transplanted orthotopically into the brain of zebrafish juveniles. As a proof of principle that these models can be used to assess drug efficacy, ERBB2-driven gliomas were successfully inhibited by treating zebrafish with a cytotoxic chemotherapeutic agent (5-fluorouracil) or a tyrosine kinase inhibitor.^71^ When feasible, orthotopic xenograft models in zebrafish brain tissue offer the advantage of more faithfully replicating human disease by utilizing the same anatomical tumor microenvironment.

### Pancreatic cancer

Xenotransplantation of pancreatic cancer cells in zebrafish has also been proposed for the screening of new anti-cancer compounds. Guo and colleagues established a pancreatic adenocarcinoma xenograft model in zebrafish embryos and adults and found that a known small molecule inhibitor, U0126, targeting the KRAS signaling pathway, represses proliferation and migration of the transplanted cancer cells in zebrafish larvae.^77^ These results suggest this model could be used to identify new therapies for pancreatic cancer.

### Retinoblastoma

Two studies have shown an orthotopic transplantation zebrafish model may represent a powerful tool for the development of specific drugs for the treatment of retinoblastoma, the most common intraocular childhood cancer, which often invades the brain and metastasizes.^61,62^ Injection of retinoblastoma cells into the vitreous cavity of the zebrafish embryo has permitted quantitative analysis of the tumor cells’ proliferative potential and the anti-cancer effect of systemically administered drugs. This model offers a potential screening platform for retinoblastoma anti-cancer drugs.^62^

### Patient-derived transplantation models

Xenotransplantation of human cancer cells directly derived from individual patients (patient-derived xenograft, PDX) represents a fascinating and forthcoming opportunity for the development of zebrafish-based patient-specific clinical approaches for cancer treatment. Such patient-derived xenografts in zebrafish offer a platform for real-time in vivo evaluation of patient prognosis and drug responses, aimed at identifying the most appropriate individualized therapy (Fig. 1, right). Although only several examples of direct transplantation of patient-derived cancer cells in zebrafish have been reported thus far,^6,8^ the rapidly increasing number of zebrafish xenograft cancer models suggests that zebrafish xenografts are on the road to a clinical application in precision oncology.

Welker and colleagues standardized a patient-derived orthotopic zebrafish xenograft model of glioblastoma. They transplanted two patient-derived glioblastoma cell lines, serum-grown adherent and neurospheres, into the midbrain region of embryonic zebrafish. In vivo tumor growth and cancer cell proliferation, migration, and differentiation were described, with different characteristics in adherent and neurosphere glioblastoma cell lines. Furthermore, currently used glioblastoma therapeutics decreased xenotransplant tumor burden and significantly rescued survival. These results provide proof of principle for the use of the model as a platform for drug screening.^9^

**Fig. 2:**
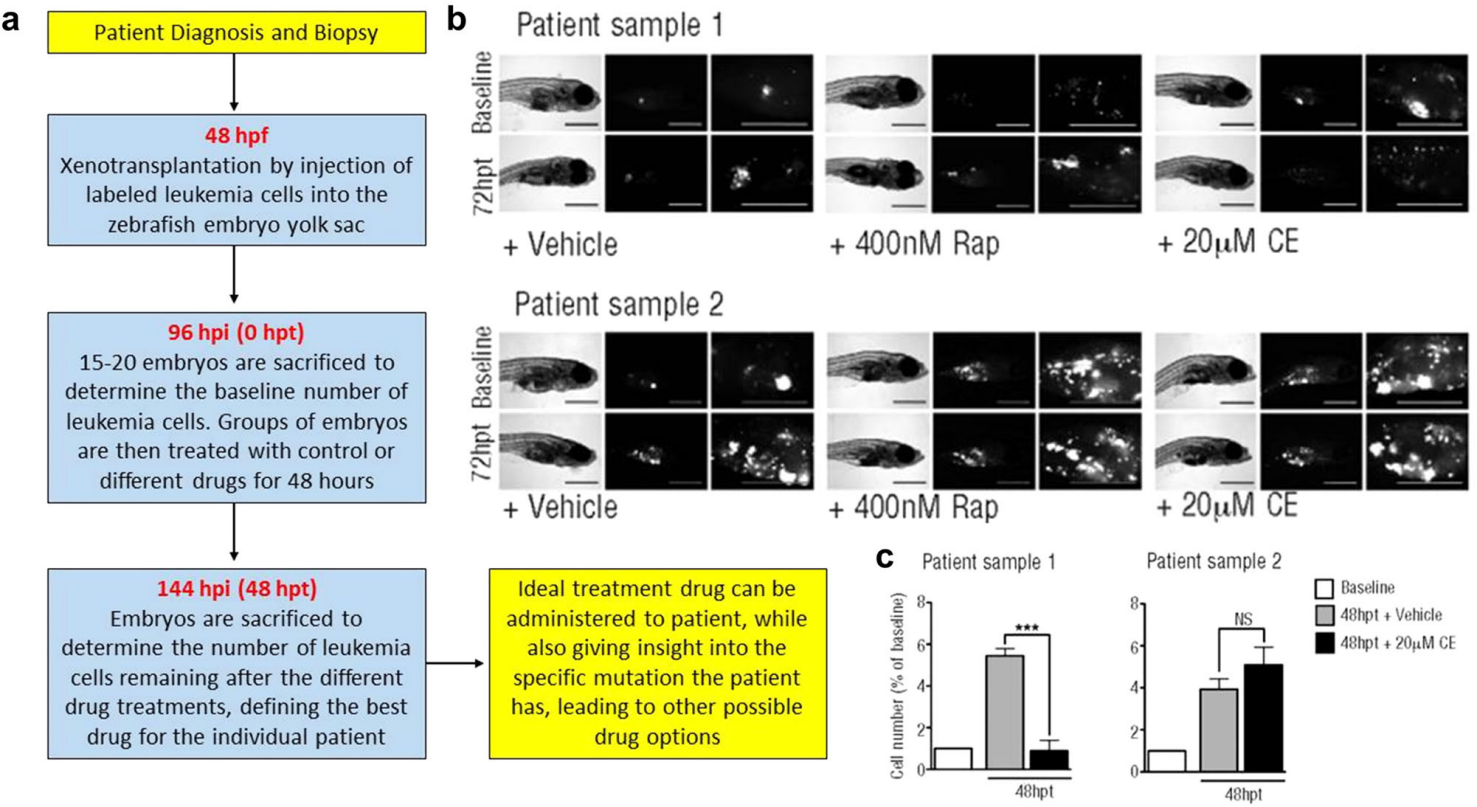
Precision oncology approach to leukemia drug screening using zebrafish. **a** Flow chart demonstrating the timeline used. Patient-derived leukemia cells were xenotransplanted into zebrafish embryos, which were administered various drugs. Leukemia cell number was used to assess drug efficacy in the zebrafish avatar corresponding to an individual leukemia patient. Assessment of drug efficacy is completed within 8 days, leading to a fast, effective, and individualized cancer treatment. hpf: hours post-fertilization, hpi: hours post-injection of cells, hpt: hours post-treatment. **b** Bright-field and fluorescence images of zebrafish injected with patient-derived leukemia cells. Embryos were treated with vehicle (control), Rapamycin (Rap) or Compound E (CE). Images were taken at 72 hpt. Scale bars are 500 µM. **c** A baseline number of leukemia cells was determined at 96 hpi. An increase in the number of leukemia cells when compared to the baseline data demonstrates cell proliferation in the zebrafish model. In patient sample one, data demonstrates a significant (*p* < 0.0001) response to the Notch inhibition (CE). The patient sample was subsequently sequenced and a gain of function mutation in the Notch pathway was found. Patient sample two did not demonstrate significant results, suggesting the mutation was not in the Notch pathway, which was subsequently confirmed through sequencing. Reproduced with permission and adapted from: Bentley, V.L. et al. *Haematologica* 100, 70–76 (2015)^5^

A preclinical human cancer xenotransplantation platform has been recently developed in zebrafish to inform therapeutic decisions in T-ALL patients (Fig. 2a).^5^ The authors previously tested the in vitro sensitivity of three T-ALL cell lines, with specific mutations in *PTEN* and *NOTCH1* genes, to three different inhibitors (targeting mTOR, AKT, and NOTCH1) and demonstrated that the same cell lines were sensitive to the same drugs upon xenotransplantation in the zebrafish embryos. The relevance of the zebrafish xenotransplantation model as a preclinical platform for a personalized therapy was demonstrated by xenotransplanting two primary patient-derived bone marrow samples into zebrafish embryos and treating with the three inhibitors. One patient sample responded drastically to NOTCH1 inhibitor (Fig. 2b, c), suggesting a mutation in the NOTCH pathway, which was subsequently confirmed to be a *NOTCH1* mutation prevalent in T-ALL. The ability to assess a patient’s responsiveness to such a targeted treatment in a zebrafish avatar (i.e., likeness, surrogate or embodiment of an individual) within 1 week following biopsy, highlights how the zebrafish xenotransplantation response can direct personalized therapy in real-time.^5^

## Transplantation metastasis models

Most cancer deaths are caused by metastasis, as opposed to primary tumors. Metastases result from the spread of cancer from the primary site to distant organs where new tumors form. Metastatic cancers have acquired the capacity to escape the primary malignant lesion site through intravasation into the bloodstream, migration, extravasation, and colonization of a distant site.^78^ Metastases are associated with poor prognosis due to the difficulty of treating such a complex and diffuse process. Moreover, unlike most other cancer processes, such as tumor initiation, proliferation, apoptosis, invasion etc., metastasis cannot be well modeled in vitro and the development and utilization of in vivo models of the dynamic sequence of steps from the local invasion to the distant colonization remains challenging. The transparency and ease of genetic manipulation of zebrafish embryos, coupled with the emerging opportunities offered by the xenograft models, represents an exceptional frontier to model and visualize the entire process of metastasis at single-cell resolution.

The xenotransplantion procedure is well optimized and automated quantitative assays are available to study invasion and metastasis of cancer cells.^79,80^ Zebrafish xenograft models of human cancer cell invasion, metastasis, and responsiveness to pharmacological or genetic intervention have been correlated to tumorigenicity of analogous human tumor cells in mouse xenograft models.^79,81^ Furthermore, Tulotta et al.^82^ demonstrated cross communication between zebrafish and human ligands and receptors, which enables the study of the interactions between human cancer cells and host microenvironment during the metastatic processes.

**Fig. 3:**
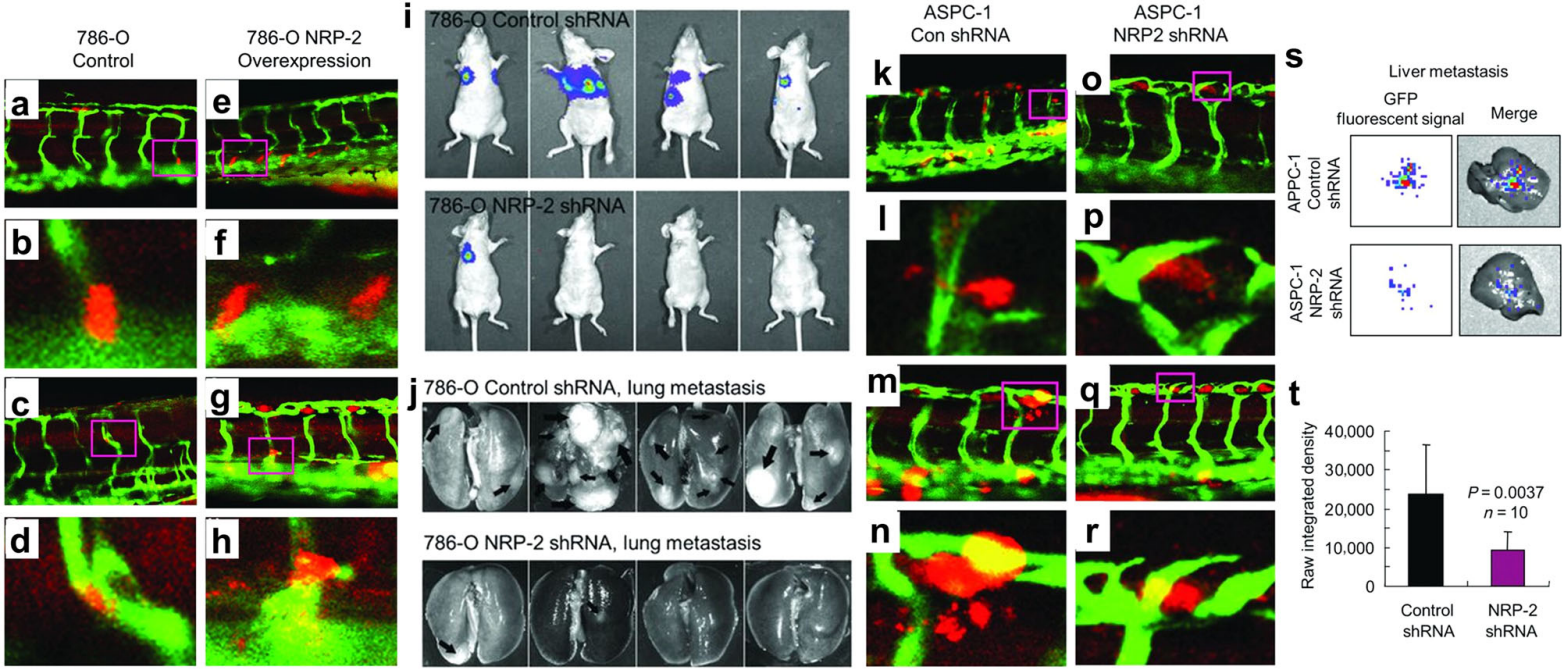
Human cancer cell xenograft models of extravasation in zebrafish and metastasis in mice. **a**–**h** Human 786-O renal cell carcinoma cells overexpressing retroviral control vector (**a**–**d**) or neuropilin-2 (NRP-2; **e**–**h**) were transiently labeled with cell tracker orange dye, microinjected into the pericardium of 3 dpf Tg(Fli-GFP) zebrafish, and imaged 1 day later. **a**–**d** control 786-O cells stay in the ISVs. **e**, **f** 786-O cells overexpressing NRP-2 extravasate from the ISVs. **i**–**j** 2 × 10^6^ luciferase-labeled 786-O cells suspended in PBS were subcutaneously injected into the right flank of female nude mice. Prior to the tumor growing to 10% of body weight, the subcutaneous tumors were surgically resected. Luciferase imaging was performed on the mice for 4–6 months to monitor metastasis, and the 786-O NRP-2 knockdown group (top) exhibited significantly fewer lung metastases than the control cohort (bottom). **k**–**r** Human ASPC-1 pancreatic cancer cells were transduced with control shRNA (**k**–**n**) or NRP-2 shRNA (**o**–**r**), transiently labeled, microinjected, and imaged as described above. **k**, **l** Extravasated control shRNA ASPC-1 cells. **m**, **l** Actively extravasating control shRNA ASPC-1 cells. **o**–**r** NRP-2 knockdown ASPC-1 cells stay in the ISV. **s**–**t** Male SCID mice were orthotopically injected with 2 × 10^6^ GFP-labeled ASPC-1 pancreatic cancer cells suspended in PBS, and after 15 days liver metastases were assessed by xenogen imaging. Reproduced with permission and adapted from: Cao Y. et al. *Cancer Res* 73, 4579–4590 (2013)^84^

Outstanding examples of zebrafish metastasis models have recently emerged (Table 3). Stoletov et al.^83^ used real-time intravital imaging to study the dynamic process of intravascular locomotion and extravasation of fluorescent human cancer cells injected into the pericardium of 48 hpf zebrafish embryos, thus providing new insights into the underlying molecular regulation, which involves β1 integrin, *Twist* and *VEGFA*. Au et al.^10^ elegantly showed the migration dynamics of clusters of circulating tumor cells isolated from the blood of breast cancer and melanoma patients. The zebrafish xenograft metastasis model represents a valuable model to test the metastatic potential of human precision oncology target genes. We recently adapted Stoletov’s model to demonstrate neuropilin-2 promotes extravasation and metastasis of human pancreatic cancer and renal cell carcinoma cells in zebrafish (Fig. 3) by interacting with endothelial α5 integrin. We demonstrate synergy of the zebrafish extravasation model with mammalian metastasis models by also exhibiting the metastatic potential of neuropilin-2 in mice (Fig. 3).^84^ Thus, zebrafish metastasis models serve as an excellent in vivo platform for validating and supporting murine metastasis data and vice versa. Our data from a separate zebrafish study revealed knockdown of a metastasis suppressor gene, non-metastatic 2 (*NME2*), promotes extravasation of A549 human lung cancer cells.^85^ In conjunction with studies of tumor transcriptomes, survival data, and prevalence of lymph node metastases in human lung cancer patients, we demonstrated that NME2 decreases metastatic potential through transcriptional repression of focal adhesion factor vinculin.^85^ In a study highlighting the mechanism by which T-lymphoblastic lymphoma progresses to the metastatic T-ALL, Feng et al.^86^ focused on the intravasation process as one of the first steps in metastasis, identifying the role of Sphingosine-1-phosphate receptor 1 (S1P1) and intracellular adhesion molecule 1 (ICAM1) in counteracting cancer cells from entering the vasculature. In a xenograft model of PDAC metastasis, the calcium binding protein S100P was shown to facilitate cancer cell intravasation and extravasation.^87^ Thus, various stages of cancer metastasis have been accurately modeled in zebrafish transplantation settings.

Zebrafish metastasis models have emerged as a promising system and continued development is likely necessary for applications in personalized medicine. Nevertheless, these models have already demonstrated their usefulness. They facilitate the investigation of new molecules shown to regulate various aspects of the metastasis process and aid in the discovery of targeted drugs that will potentiate our capacity to counteract metastasis in specific pathological conditions. For instance, Ghotra and colleagues have identified SYK as a candidate kinase target for the treatment of advanced prostate cancer. SYK was found to be upregulated in human prostate cancer and associated with malignant progression. They then used a zebrafish xenograft model to show that pharmacologic inhibitors of SYK kinase, currently in phase I–II trials for other indications, prevent metastatic dissemination of prostate cancer cells.^88^ A zebrafish xenograft model was also used to demonstrate the anti-metastatic activity of the small molecule VPC-18005, targeting the DNA-binding ETS domain of ERG, against prostate cancer cells aberrantly expressing ERG.^89^ Liekens et al.^90^ proposed the FDA-approved anti-DNA virus agent cidofovir as an anti-metastatic agent based on its ability to inhibit metastasis of FGF2-driven tumor cells in zebrafish embryos and mice. Amplification of *ADP-ribosylation factor 1* (*ARF1*) is associated with poor outcomes of patients with breast cancer. A zebrafish xenograft model demonstrated that specific inhibition of Arf1 by small molecule LM11 impairs metastatic capability of breast cancer cells. LM11 may, therefore, represent a potential precision oncology treatment for patients with *ARF1* amplification.^91^ Similarly, a zebrafish xenograft model was used to show the anti-metastatic effect of novel compounds designed to antagonize P2 × 7 receptor, previously reported as a key mediator in cancer metastasis. Significant inhibition of the invasion of MDA-MB-231 triple-negative breast cancer cells has been reported, thus identifying new potential drugs for individualized therapy.^92^ SIRT1 and CXCR4 were identified as novel pro-metastatic players, respectively, in Ewing sarcoma and triple-negative breast cancer, and the anti-metastasis effect of pharmacologically antagonizing these targets was demonstrated in zebrafish.^82,93^ Continued development of these zebrafish metastasis models, as well as establishment of new zebrafish-based systems, will accelerate their implementation in precision oncology applications.

### Patient-derived transplantation models of metastasis

Zebrafish are amenable to modeling the metastatic potential of cancer cells derived from patient specimens and transplanted in the embryo. Initial progress was demonstrated by Marques et al.^6^ by transplanting direct explants from gastrointestinal human tumors into zebrafish embryos and larvae, into the yolk sac and organotopically in the liver, respectively. They showed the zebrafish model permits rapid analysis (micrometastasis formed within 24 h after transplantation) of primary human tumor specimen metastasis. Weiss and colleagues similarly transplanted pancreatic carcinoma cells and resected specimens of human pancreatic carcinoma into zebrafish embryos. The model was used to demonstrate the anti-metastatic in vivo activity of retinoid acid receptor antagonists, following the identification of the retinoid acid target miR-10A as a key mediator of metastasis in pancreatic cancer.^8^ The possibility of a near future application of the zebrafish model in precision oncology emerges from the study of Mercatali et al.^7^ They injected zebrafish embryos with a primary culture of bone metastasis derived from a 67 years old patient with breast cancer and compared its metastatic potential with that of established cancer cell lines. Importantly, primary cell behavior reflected the clinical course of the patient’s medical history, underscoring the noteworthy benefits that such an approach might signify for the evaluation of the patient prognosis and the identification of the most appropriate individualized therapy.

## Genome editing

The advent of genome editing presents another promising strategy by which zebrafish will likely be utilized to tailor cancer therapies to individual patients. Whole-genome sequencing is commonly used to determine the molecular and genetic signature of a specific patient’s tumor. Upon identification of actionable mutations and alterations, transcription activator-like effector nucleases (TALENs) or clustered regularly interspaced short palindromic repeats (CRISPR) and CRISPR-associated systems (Cas) genome editing tools could be used to mimic key oncogenic mutations/alternations in a zebrafish avatar of an individual patient’s disease. Ekker and colleagues have used TALENs to achieve precise locus-specific DNA breaks in somatic and germline tissue of zebrafish as well as in vivo targeted knock-ins through homology directed repair.^94^ Similarly, the CRISPR/Cas9 system has been shown to be a simple, quick, and scalable technique for in vivo editing of zebrafish genes.^95^ Thus, the genome editing toolbox offers strategies for recreating oncogenic mutations and perhaps even mimicking chromosomal rearrangements or fusions through knock-in technology. Genome editing in zebrafish can be achieved in a matter of months, which makes it possible to create a genome edited zebrafish that faithfully replicates key drivers of an individual cancer patient’s tumor, in sufficient time to use the zebrafish to identify the ideal individualized treatment strategy. A similar precision medicine approach using genome edited zebrafish has been proposed to treat cardiovascular disease.^96^ Taken together, genome editing in zebrafish offers an efficient strategy for modeling the key molecular characteristics driving tumor progression in a specific patient and screening drugs to identify the ideal treatment approach in real-time.

## Challenges and shortcomings

The majority of zebrafish xenotransplantation studies require the injection of cancer cells into embryos. While this methodology has advantages as described (i.e., high-throughput injections, immunosuppression is not required, translucent embryos are amenable to in vivo fluorescent imaging, etc.), shortcomings of this approach must also be considered. The modeled tumors typically initiate during in adulthood naturally, and the embryonic environment presents undeniable differences that may impact cancer biology. For instance, embryonic development relies on the activation of specific programs and signaling pathways that are not active in adult organs under physiological conditions, but can become aberrantly activated during pathological stress, such as tumor initiation, progression, and metastasis. Various studies have utilized xenografts in adult zebrafish^69,77,86^ to overcome these concerns, but additional time and optimization is necessary for adult xenograft models to become as widely used and standardized as embryonic xenograft platforms.

Another limitation of modeling human cancer in zebrafish embryos is that such studies ignore the contribution of the immune system on cancer cell behavior, as embryos have not yet developed a functional immune system.^58,59^ This is also a drawback of human cancer cell xenotransplantation using adult zebrafish, which requires previous immunosuppression or the use of genetically immunocompromised zebrafish lines.^65,69,77,86^ A few attempts to develop immunocompetent xenograft systems have been reported. Cancers arising in syngeneic donors can be directly transplanted into a sibling recipient without the need of irradiation or dexamethasone pre-conditioning. However, only tumors made in clonal zebrafish lines can be transplanted into related recipients, and syngeneic fish are difficult to rear and not widely available.^59,97^ The impact of limitations that arise from using immunocompromised zebrafish models can be lessened by validating results in immunocompetent genetic cancer models established in adult zebrafish or other model organisms.

Long-term study of transplanted tumor cell behavior and drug response is particularly challenging. Embryo xenograft models are used in short-term assays, as long-term approaches compromise host viability. Xenotransplantation in adult fish after irradiation also prevents long-term studies because the immune system recovers within 20 days of irradiation. Dexamethasone-mediated chemical ablation of the immune system is a solution effective for solid tumor transplantation but not for leukemia. Nevertheless, mutant zebrafish lines harboring mutations that eliminate the immune response are emerging and represent a promising evolution toward the goal of long-term cancer biology and precision oncology studies in adult zebrafish.^59,97^

A recent report described a novel protocol for the long-term orthotopic transplantation of zebrafish brain tumor tissue into immunocompetent recipients. The method is based on the injection of zebrafish brain tumor cells into the fourth ventricle of a 48 hpf embryo. This allows tumors to grow in immunocompetent animals over the life of the zebrafish, enabling long-term monitoring of tumor cell behavior and drug response, including re-transplantation of tumors over many generations for potential studies on tumor evolution or drug relapse mechanisms.^97^

Lastly, a minor but intrinsic limitation of zebrafish xenografts of human tumor cells is the contrasting temperatures of the host (human cancer cells) and receipt (zebrafish). Zebrafish are typically reared at 28–29 °C, while human cells thrive at 37 °C. The compromise usually adopted is to raise zebrafish embryos at 33–35 °C following xenotransplantation of human cancer cells. Importantly, a possible impact of such temperature difference on the physiology of the fish and the biology of the tumor cells cannot be ruled out.

## Conclusions and future directions

Given the value of zebrafish as a disease model, its power as a tool in precision oncology is becoming increasingly appreciated and applications are dawning. This advancement has been fueled by studies utilizing zebrafish cancer models to define molecular players of carcinogenesis, identify actionable alterations, and develop targeted therapies. Results to date provide a basis for individualized medicine research directions as well as platforms, workflows, and systems for applications in precision oncology (Table 1).

As the number of zebrafish cancer models being developed continues to grow with a greater focus on translation studies, we anticipate zebrafish models to increasingly contribute to personalized medicine. Indeed, studies have already presented zebrafish models of human cancer with specific genetic alterations and have demonstrated models’ abilities to test and identify targeted drugs that inhibit tumor growth and metastatic potential by acting on the specific molecules and pathways responsible for the tumorigenic phenotype. One can easily visualize this approach extending to wide potentialities for defining individualized treatments for oncology patients. Given a specific tumor, driven by different mutations/alterations in different patients, which, therefore, present different responses to cancer treatment, we might imagine a tumor-specific panel of zebrafish mutants/transgenics reproducing the array of the known oncogenic alterations found in patients harboring that tumor type. A future effort to consolidate and standardize the existing models, enriching the panel with models of other significant alterations found in patients, could lead to a complete in vivo platform used to identify the best available drug for each molecular signature. Concurrently, the panel could be used to screen drugs libraries for the identification of new active compounds. Ultimately, such a strategy will define a personalized treatment plan for each patient based on the molecular, genetic, and clinical characteristics of their cancer.

A more extensive use of patient-derived xenografts in zebrafish will also represent in the near future a powerful complementary approach to develop individualized treatments and to define the most appropriate therapeutic strategy for specific alterations found in patients. First insights into the possibility of a direct, real-time application of zebrafish xenograft models in the clinic^5^ suggest a precision oncology future in which primary specimens from patients diagnosed with cancer could be xenotransplanted in zebrafish embryos to test the responses of the patient cancer cells to various available drugs. The output of the test, obtainable in days, will dictate the most effective treatment for an individual cancer patient.

Taken together, the existing and developing array of zebrafish models within the collective toolbox of the zebrafish and precision oncology research community create a promising future where zebrafish may emerge alongside current clinical applications, such genomic technologies and molecular diagnostics, to improve our ability to precisely tailor individualized cancer therapies and positively impact the clinical outcome of each patient afflicted with cancer.

### Data availability

Data sharing is not applicable to this article as no data sets were generated.
